# Improving Neonatal Care with Technology

**DOI:** 10.3389/fped.2017.00110

**Published:** 2017-05-15

**Authors:** Arjan B. te Pas

**Affiliations:** ^1^Division of Neonatology, Department of Pediatrics, Leiden University Medical Center, Leiden, Netherlands

**Keywords:** preterm infant, technology, computers, automation, therapy, computer-assisted

The care for preterm infants has improved considerably in the last decades and although the outcome improved, prematurity is still a large global health issue and is ranked in the top 10 of the WHO list of leading causes of burden of disease (1). Prematurity still leads to organ injury, especially of the lungs and brain, and is responsible for 50% of perinatal mortality (2). Historically, much progress in neonatal care was made through the use of technology. The chances of survival for preterm infants increased by the development of an infant incubator. Later on, preterm infants were able to survive by the use of a mechanical ventilator specifically designed for neonates. However, preterm infants on mechanical ventilation developed classic bronchopulmonary dysplasia, indicating that exposing fragile patients to technology can also have negative consequences. For this reason, the technology in neonatology is in a continuous process of improvement. Preterm infants are now protected by a double-walled servo-controlled incubator, and more sophisticated mechanical ventilators are used with built-in algorithms guaranteeing that even the smallest tidal volumes can be delivered in a safe manner.

Currently, technology combined with computer intelligence can support the caregiver in their daily task. Neonatal caregivers are aware that preterm infants are a very fragile patient population and that they work daily with only narrow ranges of normal and safe values, and injury can easily occur when the infant is outside the margins. Technical devices with built-in algorithms will help caregivers to maintain preterm infants within these ranges. Thus, minimizing the occurrence and duration of injurious moments can be very challenging. There is an ongoing development of technical applications specially designed to solve neonatal problems, some examples are discussed below.

## Stabilizing Preterm Infants While Cord Intact

There is now convincing evidence demonstrating that preterm infants are most vulnerable and uniquely susceptible for injury immediately after birth (3). Although neonatal care of preterm infants in the NICU has improved considerably and reduced mortality, improvements during the first 10 min of newborn life (i.e., in the delivery room) lags behind and is considered to be a large risk factor for future complications (3, 4). Not only improper ventilation can lead to lung and cerebral injury, it has become clear that immediate cord clamping, necessary to apply respiratory support as soon as possible, is also responsible for hemodynamic instability (5). Animal experiments have demonstrated that deferring cord clamping until after ventilation onset sustains preload and cardiac output and avoids the large disturbances in systemic and cerebral hemodynamic during transition (6). Efforts are now undertaken to stabilize the preterm infant close to the mother while the cord remains intact. However, it remains challenging to provide adequate respiratory support while there is no stretch or kinking of the, sometimes, very short umbilical cord of the preterm infant (7, 8). Purpose-built resuscitation tables are now developed with a platform that can be placed very close to the birth canal, making it possible to the neonatal caregiver to provide full standard care while the cord remains intact. For a successful change in the approach of preterm infants at birth, the neonatal and obstetric team will need to work together more closely and also need to be trained in the new approach. Although most projects are still in a phase to demonstrate the safety and feasibility, stabilization of preterm infants while the cord remains intact could guarantee a gentler and stable transition.

## Intelligent Use of Parameters in the NICU: Predictive Monitoring

During admission in the NICU, many data points and parameters are collected and centrally managed. These data comprise continuous measurements, such as physiological parameters (vital parameters), sensor readings, data of devices (e.g., ventilators, infusion pumps), and observations and interventions of the caregivers and results of tests (e.g., lab values, X-rays) are also noted. The high volume of data generated in an NICU with a velocity of hundreds of data points per patient-minute can be used for predictive monitoring. This involves analyzing physiological data to identify infants at high risk and detect illness in an early stage (9).

Currently, potential life-threatening complications (sepsis and necrotizing enterocolitis) are often not diagnosed until the infant’s condition is significantly compromised (9). Continuously analyzing ECG for low heart rate variability and transient decelerations to compute a heart rate characteristics index (HeRO) has been developed for early detection or prediction of sepsis (9). A multicentre randomized clinical trial in preterm infants has shown to decrease the mortality significantly (10). There is ongoing research to combine various other parameters to further improve the algorithms for early sepsis detection. Current patient monitors in the NICU have channels to incorporate the recording of ECG for heart rate, respiratory rate, airflow through the nose, oxygen saturation over time, invasive arterial blood pressure, and temperature of babies. Attempts have been made to use parameters in detecting and predicting apneas of the preterm infant (11). It is clear that there are still challenges in predictive monitoring and caregivers should be trained in how they should involve this into their clinical judgment. However, the early detection of abnormal physiological patterns has the potential to treat life-threatening complication earlier and thus improve outcomes. In addition, collecting the physiological data will become easier and less cumbersome for infants as progress has been made in contactless monitoring using photo-plethysmography, thermography, or even ultrasonography.

## Apnea of Prematurity

Apnea of prematurity, often combined with hypoxia and/or bradycardia, is one of the most common and recurrent problems in preterm infants (12). Frequent apneic spells can lead to serious brain damage and affects neurodevelopmental outcome (13). Despite great efforts to diminish the frequency and duration of apneas, frequent intervention (extra oxygen, tactile stimulation) of the caregiver is still needed in a proportion of infants (14). However, the duration of the apnea and the concomitant hypoxia and/or bradycardia depends on the response time of the nurse. Heavy workload and alarm fatigue have a negative effect on this response time (12). These issues can be overcome when the tactile stimulation (and/or increasing the oxygen) is applied automatically. Studies have demonstrated that automatic vibrotactile stimulation decreased the occurrence and duration of apneas in preterm infants (15). However, further research is still needed and devices for automatic stimulation are not yet available for clinical use. Further studies are needed to define the optimal frequency and location to apply stimulation. Also, the possible consequences of automatic stimulation on sleeping patterns needs to be further determined (16).

## Oxygen Targeting

During the admission in the NICU, preterm infants often receive supplemental oxygen for a prolonged period. Unfortunately, supplemental oxygen therapy has a very narrow therapeutic range for preterm infants and they are frequently exposed to hypoxemia or hyperoxaemia (12). To prevent this, nurses usually titrate oxygen manually to maintain SpO_2_ between the prescribed target ranges. Titrating the oxygen to keep an infant within narrow oxygen saturation target ranges can be challenging for a nurse, especially during and after an apnea accompanied with bradycardia and desaturation. Automated regulation of FiO_2_ is a technology that is increasingly used in neonatal intensive care units, and currently, trials are undertaken to test the effect on long-term outcome (17). Although studies have shown that automatic FiO_2_ reduces the occurrence and duration of hyperoxaemia, it seems to have little effect in reducing hypoxemia (12). It is possible that algorithms need to be improved as they were initially designed to prevent hyperoxemia when overshoot occurs when the oxygen is increased. Several automated oxygen control devices are now on the market and studies are needed to compare their effectiveness. However, it is obvious that automatically increasing oxygen will have no effect when oxygen desaturation is the result of an apnea. For this reason, future developments in technology could focus on an integrated automatic response in not only oxygen but also non-invasive ventilation.

## Artificial Placenta’s

The aim of an artificial placenta is to provide an environment where the fetus can continue to develop as if remaining in the maternal uterus without the physiological stress of preterm birth. The principle of treating a preterm infant as a fetus rather than a neonate is not a new idea in neonatal care (18). Studies with preterm lambs were performed since 1958 to investigate artificial placenta as a potential alternative to mechanical ventilation, and in the years, the time of survival has increased from a few hours to almost 10 days (19). The challenge lies in maintaining the diverse functions (gas exchange, hemodynamics, fluid and electrolyte balance, and renal and endocrine support) and also in maintaining the fetal circulatory configuration with patency of the major fetal shunts. However, much progress has been made in the more recent years, and researchers in the field expect to have the first studies in extreme preterm infants within 5 years (20). Implementing the artificial placenta into clinical practice would definitely be a new milestone in neonatal medicine.

These were just a few examples, but it is clear that clinical problems related to preterm birth can be approached by the use of technology and computer intelligence. The possibility to have continuous surveillance and development of algorithms would help the neonatal caregiver to keep the preterm infant within its narrow therapeutic ranges and limits of safety. Using technology in an adequate manner has the potential to decrease the burden of preterm birth and reduce health costs. However, nature is complex, and it will be a big challenge to improve the applied technology to deal with this complexity. It will also be a big challenge for neonatologist to keep up as these developments in technology can go fast. To use it properly next to their clinical judgment, neonatologist should understand how the technology works and recognize malfunction and/or artifacts. Perhaps in the future, it will be necessary for neonatologists to also receive training in the technology used, next to their medical training. Parallel to this, a technical engineer could be added to the neonatal team and attend the daily rounds. Last but not least, with the increasing possibilities, technology should be used wisely and appropriately, and the potential benefit should always be weighed against the possible harm/burden. The neonatologist should be at the center of debate concerning the limits in applying this technology.

## Author Contributions

The author confirms being the sole contributor of this work and approved it for publication.

## Conflict of Interest Statement

The author declares that the research was conducted in the absence of any commercial or financial relationships that could be construed as a potential conflict of interest.
